# Cow’s Milk Processing—Friend or Foe in Food Allergy?

**DOI:** 10.3390/foods10030572

**Published:** 2021-03-09

**Authors:** Sabine Geiselhart, Aleksandra Podzhilkova, Karin Hoffmann-Sommergruber

**Affiliations:** Department of Pathophysiology and Allergy Research, Medical University of Vienna, 1090 Vienna, Austria; sabine.geiselhart@muv.ac.at (S.G.); aleksandra.podzhilkova@muv.ac.at (A.P.)

**Keywords:** cow’s milk allergens, whey, casein, food processing, IgE-mediated food allergy

## Abstract

Cow’s milk (CM) is an integral part of our daily diet starting in infancy and continuing throughout our lifetime. Its composition is rich in proteins with a high nutritional value, bioactive components, milk minerals including calcium, and a range of immunoactive substances. However, cow’s milk can also induce a range of immune-mediated diseases including non-IgE-mediated food allergies and IgE-mediated food allergies. Cow’s milk allergens have been identified and characterized and the most relevant ones can be assigned to both, the whey and casein fraction. For preservation a range of processing methods are applied to make cow’s milk and dairy products safe for consumers. However, these methods affect milk components and thus alter the overall immunogenic activity of cow’s milk. This review summarizes the current knowledge on cow’s milk allergens and immunoactive substances and the impact of the different processes up- or downregulating the immunogenicity of the respective proteins. It highlights the gaps of knowledge of the related disease mechanisms and the still unidentified beneficial immunomodulating compounds of cow’s milk.

## 1. Introduction

Milk consumption by humans has been tightly connected with settledness and agriculture from the very beginning. Recent findings provide evidence that humans were already drinking milk at least 6000 years ago [1]. Collecting, processing, and consuming milk from animals enabled one of the most profound revolutions in human diet and for centuries, cow’s milk has been an integral part of our diet. Animal milk is a renewable food source rich in proteins, fat, and micronutrients. For centuries, it has been used as alternative for breast milk and enabled early weaning with significant demographic implications. Since then strategies for preserving milk have been developed and continuously improved—for example, the generation of yogurt, butter, and cheese are old traditional methods. Nowadays, highly refined technologies are in place to offer safe and convenient consumption. However, these methods may also have a negative effect on the micronutrients which are present in natural milk products. Furthermore, the food and pharmaceuticals industries use individual milk components in many different applications.

It is also known that milk can induce immune-mediated diseases. Although milk is regarded as a healthy food with high nutritional value it can be harmful to some individuals with a predisposition to develop immune-mediated adverse reactions to foods. Food allergy prevalence rates have increased, including cow’s milk allergy. Changes in lifestyle and dietary habits may account for this, since milk consumption is no longer only regarded as part of a healthy diet for infants and children—nowadays it is consumed throughout one’s lifetime. Moreover, exposure to milk-derived components (milk protein, sugars, lipids) has increased since they are present in a range of highly processed convenience food products, cosmetics, and pharmaceutical drugs.

This review is dedicated to summarizing the current knowledge of milk allergens and the food processing techniques which may modulate their immunogenicity.

## 2. Brief Overview on Immune-Mediated Diseases Caused by Milk Proteins

### 2.1. IgE- and Cell-Mediated Allergies

Adverse reactions to foods can induce a variety of immune-mediated reactions, ranging from mild to severe symptoms, some of which persist throughout one’s lifetime while others resolve. For a restricted number of food-related diseases the causative foods are known, including milk and dairy products. After birth newborns are usually breastfed and in due course exposed to cow’s milk and/or related milk sources, representing one of the earliest encounters with foreign (nonself) dietary antigens. Therefore, most immune-mediated diseases caused by milk intake start early in life and resolve within early childhood, while in adolescence and adulthood only a comparatively reduced number of diseases persist or develop.

Adverse immune reactions to foods can be assigned to IgE-mediated, mixed IgE- and cell-mediated, and cell-mediated groups based on the underlying mechanisms [2]. Currently, food protein-induced enterocolitis syndrome (FPIES), food protein-induced enteropathy (FPE), and food protein-induced allergic proctocolitis (FPIAP) are listed under “non-IgE-mediated gastrointestinal food allergic disorders (non IgE-GI-FAs)” [3]. These diseases start early in infancy, are relatively rare and with a good prognosis resolving after 1–3 years. The majority of patients experience symptoms until the age of 5 years [3]. For these diseases cow’s milk proteins seem to be a relevant trigger, although other food sources such as soy, wheat, and egg have been reported. For all these foods no specific immunogenic proteins have been identified so far; it is generally accepted that the cow’s milk protein fraction per se is causative.

Food protein-induced allergic proctocolitis (FPIAP) is a cell-mediated immune disease caused by cow’s milk, soy, egg, and wheat in the maternal diet when breastfed, and milk and soy formulas [3]. Usually, the symptoms are bloody intermittent stools in otherwise healthy and thriving babies [3]. Although the underlying pathomechanism is not well understood, increased eosinophils have been identified in intestinal endoscopies. Diagnosis is based on characteristic symptoms and elimination diet for diagnostic purposes can be performed. After 4–8 weeks, reintroduction of the causative foods is possible based on symptom amelioration. Usually FPIAP resolves after some months.

Food protein-induced enterocolitis syndrome (FPIES) starts within the first year of life. The clinical picture includes malabsorption, anemia, diarrhea, and vomiting and can lead to failure to thrive. Similarly to FPIAP the pathogenesis of FPIES is not well understood but studies provided evidence of T cells secreting inflammatory cytokines and the role of neuroendocrine pathways in this disease was discussed. Moreover, neutrophilia and thrombocytosis are frequently identified in patients [4]. Milk was in most cases the causative food and children resolving the disease within 5 years had no detectable milk specific IgE antibodies, whereas patients with milk positive IgE had consistent milk adverse reactions [5].

Food protein enteropathy (FPE) usually presents with chronic diarrhea which does not result in severe dehydration. Cow’s milk protein is also the most important food trigger for this disease. The histology of FPE patients provides damage to the villi, and the presence of eosinophils and cow’s milk-specific Th2 cells and sometimes local IgE production.

In summary, non-IgE-GI-FAs share the same food(s) that trigger/induce symptoms, their individual pathomechanisms are not well understood and a potential risk of underdiagnosed cases is possible. However, the majority of cases experience tolerance induction within the first year [6]. The question remains whether cow’s milk proteins are the causative trigger of the diseases mentioned above or whether it is the first encounter between dietary proteins and an as yet immature digestive tract which causes the symptoms.

Another immune-mediated disease that is triggered by certain foods including milk is eosinophilic esophagitis (EoE). This disease is diagnosed by a high number of eosinophilic infiltrates and dysfunction of the esophagus resulting in difficulties swallowing food and food impaction. This fairly recently identified disease has been diagnosed in both children and adults. Although food-specific IgE antibodies have been determined in a subgroup of patients, it remains to be established if the presence of IgE antibodies is an epiphenomenon or linked with the pathomechanism which is still poorly understood for EoE [7].

### 2.2. IgE-Mediated Milk Allergy

#### 2.2.1. Prevalence

Cow’s milk is one of the first nutritional sources for infants and in atopic individuals IgE-mediated allergic symptoms can be diagnosed soon after first exposure. Symptoms range from acute cutaneous reactions e.g., urticaria, atopic dermatitis, immediate breathing problems, gastrointestinal problems, and asthma attacks up to anaphylactic reactions. In a systematic review prevalence rates of 6.0% for self-reported cow’s milk allergy were published, compared to 0.6% for an objectively verified cow’s milk allergy [8]. Higher prevalence data are known for infants and children as compared to adolescents and adults, which is due to tolerance induction in the majority of children when reaching school age.

The underlying pathomechanism of an IgE-mediated allergy is based on the production of allergen-specific IgE antibodies from B cells together with the favoring cytokine milieu provided by T helper cells type 2 in genetically predisposed individuals. When reexposure to the same allergen source occurs the allergen is recognized by IgE antibodies that are bound to receptors on mast cells and basophils and upon cross-linking an immediate reaction is triggered by the release of active substances and cytokines. So far, a number of cow’s milk allergens have been identified and their immunogenic activity can be tested in cellular tests and in vitro diagnostic tests. A detailed overview of the currently known cow’s milk allergens is presented below.

Diagnosis is based on taking patient history including reported symptoms, a skin prick test and an in vitro test for allergen-specific IgE antibodies. In addition, an oral food challenge either in an open or blinded setting can be performed. For in vitro tests the detection of serum-derived milk allergen-specific IgE is identified in assays that use either total extracts or panels of individual allergens. In the later assay the specific immune response to single allergens provides a detailed analysis of the allergens causing the immune response in each patient.

In cellular tests basophils are used and incubated with the patient’s serum-derived IgE antibodies. Upon the addition of milk allergens and/or milk protein extract cross-linking takes place, providing a positive test result.

For food challenges different protocols have been developed including the approach using “baked milk”. This approach is applied to investigate whether there is an increased risk of persisting milk allergy compared to tolerance induction to milk proteins during childhood. The underlying rationale is that heat treatment of milk proteins can affect their structure and thus their presentation of IgE epitopes. If patients tolerate baked milk whereas they react to untreated milk extract, the probability of developing a tolerance to milk products is very likely (see also Section 5.1).

#### 2.2.2. Treatmen

The avoidance of milk and dairy products is the method of choice for treatment. For infants peptide based milk formulas can be offered. These peptides or amino acid formulas are unable to trigger the cross-linking of IgE and are thus safe for consumption by those with milk allergies. For a subgroup of people with milk allergies, heat-treated milk can also be tolerated (see also heat processing and effect on allergen structures). The use of goat’s milk or sheep’s milk as an alternative nourishment to breastfeeding and cow’s milk is not recommended. Although in some cases amelioration of symptoms is observed, the effect does not last and the allergic symptoms reoccur. This is due to the presence of highly homologous allergens in the milk products of these closely related species.

As mentioned above, the majority of milk allergic children gain tolerance and upon a confirmative negative food challenge milk can then be reintroduced to the daily diet. Currently, no approved immunotherapy for milk allergy is available; however, in a number of specialized centers, a rush immunotherapy can be offered to highly sensitive patients so that they reach tolerance to minute amounts of milk proteins.

#### 2.2.3. Prevention

Regarding the prevention of IgE-mediated cow’s milk allergy, the introduction of cow’s milk into the diet between months 4–6 is recommended, followed by regular cow’s milk ingestion, based on recent studies [9].

## 3. Milk and Its Components

The milk of herbivorous species (cows, sheep, goats, etc.) comprises homologous proteins with similar structural, functional, and biological properties. Along with proteins, milk contains nitrogenous compounds of a nonprotein nature: free amino acids, peptides, urea, ammonia and uric acid, etc. Furthermore, a huge amount of bioactive components such as casein hydrolysates, lactoferrin, lactoperoxidase, lysozyme, glycomacropeptide or caseinomacropeptide, whey protein hydrolysate, milk minerals including calcium and magnesium [10], α-lactalbumin, galacto-oligosaccharide (GOS), conjugated linoleic acid (CLA), are present in milk [11]. All these constituents have a particular biological impact on human health.

**Figure 1:**
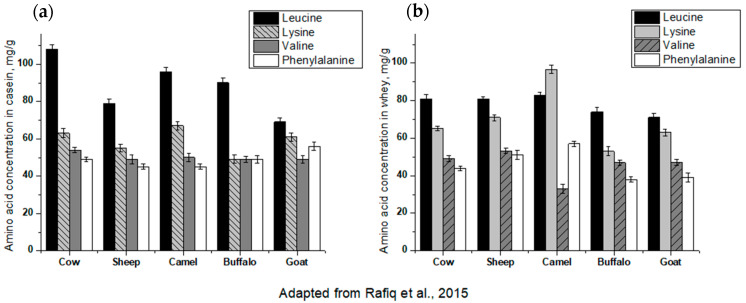
(**a**) Essential amino acids in caseins from different species. (**b**) Essential amino acids in whey from different milk species. (adapted from [14]).

**Figure 2:**
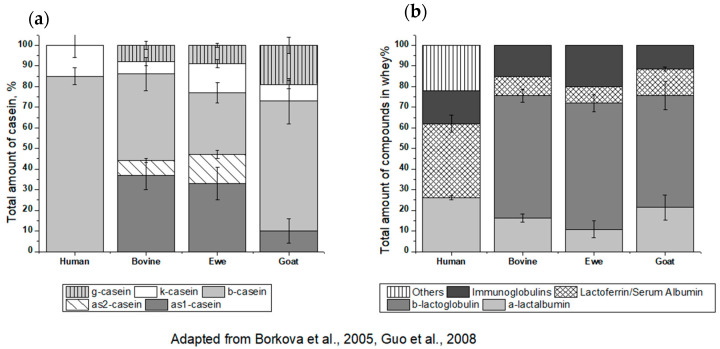
(**a**) Representation of caseins in human, raw bovine, ewe, and goat’s milk. (**b**) Composition and content of whey proteins in human, skimmed cow, ewe and goat’s milk adapted from [15,16].

Milk includes more than 40 proteins (30–35 g per liter), comprising 80% casein and 20% milk serum (whey) [12]. Cow’s milk contains allergens and homologues of those are also present in all ruminant species (Table 1). They share structural, functional and biological properties [13,14]. In addition, milk contains a range of immunoactive substances such as osteopontin, cytokines (e.g., TGF-beta, IL-10), alkaline-phosphatase and vitamin D. According to the authors, leucine is the major amino acid in casein and in whey, as shown in Figure 1 [14] and Figure 2 [15,16]. In Figure 2, caseins and whey proteins from different species are presented. As we can see, in human milk β-lactoglobulin and the caseins as1 and as2 are absent. 

## 4. Cow’s Milk Allergens

So far, eleven individual IgE-binding allergens from *Bos domesticus* have been accepted by the official allergen nomenclature committee (www.allergen.org) (accessed on 27 January 2021). Out of those, eight are present in milk (Table 1).

### 4.1. Whey

Milk whey proteins are characterized by high nutritional value, have the ability to emulsify lipids and to bind and retain water, which improves the structural and organoleptic properties of food products [18,19]. According to Monaci et al., the quality of whey proteins is well-suited to their unique amino acid composition, which is better balanced than caseins [13]. The majority of whey is globular proteins, α-lactalbumin and β-lactoglobulin, which are produced in the mammary glands. Other proteins, such as bovine serum albumin (BSA), lactoferrin and immunoglobulins derive from blood.

#### 4.1.1. Beta-Lactoglobulin (Bos d 5)

Beta-lactoglobulin (Bos d 5)-a small protein with a molecular mass of 18.3 kDa, makes up to 50% of all whey proteins and 10% of whole milk proteins but it is essentially absent in human milk [20,21]. Beta-lactoglobulin, a dimeric protein from the lipocalin family, is one of the best characterized lipid-binding proteins. In addition, the protein is also efficiently binding many hydrophobic molecules, suggesting a role in their transport [13]. It is already known that Bos d 5 is one of the major allergenic proteins in cow’s milk (Table 1). Bos d 5 contains about 8% of α-helices, 45% of β-sheets and 47% of random coils [14]. Some of the β-sheets comprise a “calyx” with two different sheets from β-A to β-D and from β-E to β-I [22]. It consists of 162 amino acids and occurs as three variants (A, B and C) with two disulfide bonds and one free sulfhydryl group buried within the protein structure [22]. These three variants A, B, and C, which were recently studied, contain two different point mutations [22]. Although the structure of the A and B variants is almost identical, they differ in amino acid residues: Asp64 in A is changed to Gly in B, and Val118 in A is changed to Ala in B [17,22]. Based on the results of the experimental studies, these two amino acid exchanges account for the different intensity in and duration of the IgE response [13]. Moreover, it was suggested, that the structure of variant A is more flexible compared to variant B [23]. However, variant B is more thermally stable than variants A and C.

Under certain conditions (including pH, temperature, ionic strength and protein concentration), the individual β-lactoglobulin isoforms are present in different oligomeric states. For example, after increasing acidic conditions we can observe that genetic variant A (dimer) associates into octomers with 144 kDa, while variants B and C do not oligomerize to octomers [22]. Different conformational changes and reversible dissociations appear at 60 °C. Some irreversible conformational changes in monomers can happen after heating up to 70–80 °C.

As mentioned above, β-lactoglobulin is a major allergen, recognized by specific IgE in more than 50% of milk allergic patients. The molecule contains several IgE epitopes, which are located (exposed) on its surface. It was previously shown that patients with IgE- mediated cow’s milk allergy had seven IgE and six IgG binding epitopes, while in younger patients only three of these IgE binding epitopes were recognized [24]. In that case, a large number of β-lactoglobulin epitopes may be a marker of persistent cow’s milk allergy. While much is known about the allergenic activity of Bos d 5 less is known about unexpected exposure to milk allergens. For example, β-lactoglobulin was also detected in house dust and cosmetics [21].

Interestingly, β-lactoglobulin can be used as a transporter for drugs in cancer treatment because of the physicochemical properties of the protein and its ability to bind a wide range of different ligands [25,26,27].

#### 4.1.2. Alpha-Lactalbumin (Bos d 4)

Alpha-lactalbumin ranks second after β-lactoglobulin in whey proteins regarding abundancy. It represents 20–25% of whey proteins, or 2–5% of the total protein [14]. Alpha-lactalbumin is a 14.2 kDa (pH 4–5) monomeric globular protein [28]. The protein regulates the production of lactose in the milk of almost all mammalian species and it is found in considerable quantity in human breast milk.

Bos d 4 has two Ca^2+^ binding sites [17,20,28] and therefore many researchers use this protein as a calcium-binding model. Alpha-lactalbumin has intermediate molten folded globule-like states, which is of relevance for food processing strategies [29]. Moreover, it was recently shown that certain α-lactalbumin variants might induce apoptosis in tumors. A lower number of α-lactalbumin molecules binding oleic acid as a cofactor and thus can induce cytolysis of several types of malignant cells [18].

The primary structures of bovine and human α-lactalbumin share 72% sequence similarity. It has antibacterial and immunostimulating properties, which makes it a protein with high nutritional value in general and especially for babies [30].

Native α-lactalbumin from different species (humans, cows, camels and goats) consists of 123 amino acids. Furthermore, the protein contains a large number of essential amino acids (Trp, Val, Lys, Ile, Leu, Thr, Met, Phe and His) ensuring its excellent nutritional value. The amino acid composition is dominated (milligrams of amino acid per gram of protein) by leucine (108), lysine (109) and isoleucine (60) [31]. It should also be noted that four cysteine residues (48), allow the formation of disulfide bridges, and it can be released by digestion and appear in the blood as either the disulfide cysteine or free cysteine [31]. Bos d 4 contains two structural regions: a large α-helical domain and a small β-sheet domain, which are separated from each other by a deep cleft [29] but they are held together due to the cysteine bridges that form the Ca^2+^ binding loop. Ostrovsky et al. provided information about the structural changes of Bos d 4 after binding Ca^2+^. They observed that this binding might lead to both a tryptophan fluorescence blue shift and a decrease in fluorescence quantum yield, respectively [32]. While α-lactalbumin in cow’s milk represents up to only 25% of the whey fraction, the human α-lactalbumin increases up 40% [18] (Figure 2b).

#### 4.1.3. Serum Albumin (Bos d 6)

Serum albumin is the main protein of mammalian blood, present in milk and meat. It is present in milk up to 5% of total whey proteins (67 kDa). Bos d 6 is described as a protein with strong ligand binding capacity. It not only binds fatty acids, but also flavor compounds and metal ions [18]. As mentioned above, the concentration of Bos d 6 in milk is low, and BSA has little effect on the physicochemical properties of whey protein concentrates and whey protein isolates [33]. Bovine serum albumin contains 584 amino acids. The protein consists of nine loops connected by 17 disulfide bonds [12]. Bos d 6 is a minor allergen, affecting <50% of cow’s milk allergic patients. Interestingly, it was described that beef allergic patients sensitized to Bos d 6 develop cross-reactivity to milk [19].

Moreover, patients with milk allergy and sensitization to cow’s serum albumin are at risk of developing sensitivity to animal dandruff, which can be a cause of rhinoconjunctivitis and/or bronchial asthma [14].

### 4.2. Caseins (Bos d 8; Bos d 9–12)

Caseins (Bos d 8) form the main protein fraction of cow’s milk (80%) and consist of: αS1- (Bos d 9), αS2- (Bos d 10), β- (Bos d 11), and κ-caseins (Bos 12) representing 40%, 13%, 37%, and 10%, respectively [13].

Caseins play a significant role in human health. The biological function of caseins is to [34,35] (1) carry calcium and phosphate (preventing the calcification of the mammary gland); (2) to provide immunological protection to infants; and (3) containing high content of amino acids, minerals, and lipids [36].

It was shown [37] that caseins aggregate into particles with micellar structure with colloidal calcium phosphate in fresh milk. The molecules form a casein micelle with a hydrophobic central part and a hydrophilic peripheral layer [14]. The size of casein micelles ranges from 0.01 to 0.3 μm. This micelle formation can be applied to deliver various bioactive food ingredients [38].

Caseins are usually phosphate-conjugated, and form calcium phosphate-micelle complexes with mainly αS1-, αS2-, β- and, κ-caseins.

Alpha S1-casein is the main fraction of casein (40%) and consists of one major and one minor subunit [39]. According to Chianesea et al., bovine αs1-casein contains two common isoforms (A, B) with one amino acid exchange of Leu 178 (A) → Ser 178 (B) [40]. IgE sensitization is especially frequent against αS1-casein, inducing strong immediate or delayed allergic reactions [41].

Alpha S1-casein consists of 199 amino acids with a high amount of proline residues and a lack of disulfide bonds [42]. In that case, all IgE epitopes are linear. Cocco et al. reported that denatured α-casein can bind IgE with the same binding capacity compared to native α-casein [42].

Alpha S2-casein, representing up to 13% of the caseins in cow’s milk, is hydrophobic and the most phosphorylated casein fraction [43]. It has been shown that αS2-casein could form amyloid fibrils at 37 °C, however, only under nonreducing conditions [34].

Beta-casein represents about 35–37% (209 amino acids) of caseins. Plasmin can degrade β-casein into γ1-γ2-γ3-casein fragments [13]. Beta-casein consists of two isoforms: A1 and A2, which differ in amino acid residue 67 (Histidine in A1 and Proline in A2) [44]. Chatchatee et al. identified six major and three minor IgE-binding epitopes using sera from 15 milk allergic patients [24].

Kappa-casein represents only 10% of caseins [13]. Nine different isoforms (A to J) of κ-casein were found [45]. The main isoforms (A and B) differ at position 136 (Ile → Thr) and 148 (Ala → Asp). Kappa-casein contributes to the stability of milk due to its ability to provide steric and electrostatic repulsion [46]. So far, eight major IgE epitopes have been identified by Chatchatee et al. [24].

It has been shown that even after heating caseins do not undergo significant structural changes [13]. However, caseins are sensitive to degradation by various proteinases. Caseins have different primary structures and functional properties. For example, three of them—αS1-, αS2-, and β-caseins—are calcium-sensitive, while κ-casein is not [13].

The biological function of caseins is to transfer nutritional components from the mother to the newborn [47]. Due to their colloidal properties, they are also added to a large number of food products, cosmetics and drugs, such as infant food and protein cocktails [47].

Interestingly, caseins can be used as a carrier of different drugs and pharmaceutical compounds [47]. Gandhi and colleagues showed that casein nanoparticles with doxorubicin (1.29 μg/mg of casein nanoparticles) could release 90% of the drug doxorubicin under acidic pH. In that case, these nanoparticles can act as a drug release vehicles that enable successful drug delivery into the stomach [47].

### 4.3. IgE Cross-Reactivity of Milk Proteins from Different Species

It is well known that people with cow’s milk allergies can develop symptoms when consuming milk from other species. In a study performed by Restani et al., serum samples from patients with cow’s milk allergy were tested for IgE cross-reactivity to milk from other species [48]. It was shown that specific IgE antibodies recognized proteins from buffalo’s, goat’s, and ewe’s milk. Cross-reactivity between cow’s milk’s components and other mammalian species’ (ewe and buffalo) is evident for caseins (especially for αS1, αS2-caseins) and for β-lactoglobulin [48]. Most interestingly, children with cow’s milk allergy did not show cross-reactivity to camel’s milk. This was confirmed by another study from Ehlayel et al. showing that almost 80% of cow’s milk allergic patients tolerated camel’s milk and had negative skin-prick test results [49].

Bellioni-Businco et al. investigated, in vitro and in vivo, the allergenicity of goat’s milk in children with cow’s milk allergy [50]. The authors concluded that goat’s milk is not a recommended substitution for children with cow’s milk allergy. In another study Businco showed that 96% of children with IgE-mediated cow’s milk allergy (n = 25) tolerated consumption of mare’s milk [51].

Summarizing, camel and mare’s milk might be a promising substitute for cow’s milk for allergic children; however, these studies need to be performed in larger cohorts.

A range of different processing techniques for milk have been developed and the following chapter will provide a summary on the currently applied methods and their impact on the individual cow’s milk allergens.

## 5. Food Processing: Applied Techniques and Effect on the Allergenicity of Individual Cow’s Milk Proteins

Cow’s milk is consumed daily worldwide with annual total numbers of 81 billion tons for India, 33.4 billion tons for the EU and 21.2 billion tons for the United States in 2019 according to Statista (https://www.statista.com) (accessed on 27 January 2021). In industrialized countries fresh cow’s milk is usually extensively processed to be safe for human consumption, to meet consumer requirements and also to prolong its shelf life. After harvesting, milk is immediately cooled and transported to milk factories.

The raw milk is subjected to different processes to inactivate pathogenic microorganisms such as bacteria, spores, yeast, molds, and viruses, which can cause health problems in humans. Mostly heat-treatments are applied, although microfiltration processes are also put in place [52]. Additional processes employed include conventional techniques such as homogenization, fermentation to produce yogurts, vacuum evaporation to obtain condensed milk, or spray drying for milk powders. Newer methods such as irradiation, ultrasound processing, or cold plasma treatment are also applied.

Different treatments may affect milk proteins and induce modifications. The ratio in which the different modifications occur depends on the processes that are employed and their combination to obtain specific products (liquid and dry dairy products). Furthermore, chemical reactions occur between proteins and fat and sugars of the food matrix.

These methods can be categorized into two processing types, thermal and nonthermal. In the first case, food can be thermally processed by using moist heat or dry heat. Each of these steps induces profound changes in the quality of the milk, resulting in altered health properties.

### 5.1. Thermal Processing

Differences in time and temperature are the most crucial factors during heat-treatment. In industry, three principal categories of moist heat-processing are commonly used: pasteurization, sterilization, and ultrahigh temperature (UHT) processing.

#### 5.1.1. Pasteurization

For pasteurization, the conditions vary from 65 °C for a few seconds to more than 80 °C for up to several minutes. Even mild conditions are appropriate to destroy pathogens while other microorganisms are significantly reduced (depending on the temperature and the time) but can still cause spoilage.

The degree of the structural changes of the proteins occurring during heat-treatment depends on the thermal conditions and time of treatment as well as on the type of protein and the presence of other food components such as lipids and carbohydrates, known as the “matrix effect” [53].

Caseins are stable to heat-treatment because they show very few secondary and tertiary structures (as described above). Morisawa and colleagues showed that heat treatment alone without subsequent enzymatic digestion did not alter the allergenicity of α-casein [54]. In line with this, Bloom et al. demonstrated that when using sera from milk allergic patients, IgE binding to heat-treated casein (90 °C) persists regardless of heating time. Interestingly, the presence of wheat during heating resulted in the decreased binding of the specific IgE to milk proteins. In contrast, in a study by Xu et al., the allergenicity of α-casein and β-casein showed varied changes, but was generally lower than in the untreated samples. When heating up to 65–70 °C, the allergenicity of α-casein decreased, whereas the allergenicity of β-casein severely increased, leading to the conclusion that different proteins show different sensitivities under heat treatment [55]. A recent study in a Moroccan population on the allergenicity of caseins after heating revealed reduced binding of specific human IgE [56].

Whey proteins are unstable when heated, leading to alterations in structure and thus allergenicity. It has been shown that the antigenicity of α-lactalbumin and β-lactoglobulin increases in parallel with temperature from 50–90 °C, with the highest antigenicity of α-lactalbumin and β-lactoglobulin detected at 90 °C. However, above 90 °C the antigenicity of both proteins showed a remarkable decrease.

#### 5.1.2. Sterilization

When milk is sterilized (120 °C for 20 min), the antigenicity of α-lactalbumin decreased below the initial value of the untreated sample [57]. Heat-treatment also reduced the IgE-binding capacity of β-lactoglobulin [58]. Xu et al. obtained comparable results when heating cow’s milk allergens to 65–100 °C for up to 30 min. The antigenicity of α-lactalbumin significantly decreased, whereas that of β-lactoglobulin showed an increase up to 85 °C but decreased significantly at higher temperatures [55].

In line with this, thermal treatment of β-lactoglobulin (80–100 °C) reduced its ability to induce histamine release from sensitized human basophils [54]. Thus, it seems that β-lactoglobulin presents new epitopes upon heating, but at temperatures above 85 °C it builds aggregates via covalent and noncovalent interactions, thus masking conformational epitopes. Linear epitopes also become inaccessible in this compact structure, resulting in decreased allergenicity [59].

In vivo studies in mice revealed that oral sensitization to raw milk showed fewer acute allergic symptoms upon intradermal administration compared to processed milk. Allergen-specific IgE levels and Th2 cytokines were also significantly lower in mice sensitized to raw milk. This showed that raw milk and native whey proteins have a lower allergenic potential than their processed counterparts. These results were supported by a pilot study where milk allergic children tolerated up to 50 mL raw milk but only 8.6 mL processed “shop milk” [60].

Contradicting results came from a study in brown Norway rats where heat-treated whey proteins showed reduced intraperitoneal sensitizing capacity. Interestingly, heat-treatment did not influence the oral sensitizing capacity but significantly reduced the eliciting capacity compared to unmodified whey upon oral challenge. Heat-treatment did not reduce the tolerogenic properties of whey, as it equally prevented sensitization in naïve rats. Another interesting finding of this study was that heat-treated whey protein was less absorbed via the epithelium but more into the Peyer’s patches. The authors concluded that the route of the uptake in the digestive tract may affect protein allergenicity [61].

#### 5.1.3. Ultrahigh Temperature (UHT) Processing

UHT processing is done at higher temperatures compared to pasteurization and sterilization. During UHT, milk is exposed to a temperature of at least 135 °C to destroy bacterial and fungal spores, but only for a few seconds. As mentioned above, the heating of milk proteins leads to protein denaturation as well as extensive chemical modification (Maillard reaction). However, under these conditions the level of chemical modifications is much lower compared to lower temperatures for extended times [62], making UHT processing popular for industry. Unfortunately, little is known about the effect of UHT processing in the context of allergy. One study exists evaluating the immunogenicity of UHT treated milk by skin prick test in children with cow’s milk allergy. However, UHT treated milk does not behave significantly different from other forms of cow’s milk in this setting [63].

#### 5.1.4. Baking Milk

There is emerging evidence that the properties of allergenic proteins are more complex than being stable to heat-processing or not and that the food matrix plays an important role. The majority of milk allergic children tolerate products containing baked milk. It has been shown that milk allergenicity is changed by the baking process in muffins. Baked milk within a matrix such as wheat is less likely to cause allergic reactions [64]. It seems that major allergens are destroyed during baking. The eliciting dose in children tolerant to baked milk was also higher [65,66]. Furthermore, in a follow up study it was shown that a diet including baked milk is associated with progressive immunomodulation compared to strict avoidance of milk products [67].

#### 5.1.5. Spray Drying

Milk powder has a much longer shelf life and also lower transportation costs than liquid milk. Currently, it is produced through spray drying, where the liquid material is vaporized quickly with hot air. Lactose is a reducing sugar present in milk that under certain heating conditions reacts with free amino acid side chains, mainly from lysine, to form glycation products. These modifications have been shown to alter the allergenicity of proteins. However, little is known about the effect of spray drying on the allergenicity of milk allergens. A recent study investigated the changes of milk proteins after simulated industrial processing [68]. The authors could show that the degree of glycation after spray drying (170 °C) was increased, although only slightly. Another study investigated the IgG and IgE binding capacity of β-lactoglobulin after spray drying. At a drying temperature of 120 °C no changes were found, whereas when β-lactoglobulin was spray dried with 180 °C under the presence of lactose, aggregation occurred and 7 lysine side chains were modified and the IgG/IgE binding capacity decreased [69]. A study examining the differential effects of the dry vs. wet heating of β-lactoglobulin revealed that dry heating requires the presence of lactose to show increased IgE recognition in most individuals tested [70].

### 5.2. Nonthermal Processing

#### 5.2.1. Homogenization

Heat-treatment is often followed by homogenization to prevent phase separation, improve emulsion and also to increase shelf life. For homogenization, milk is forced through narrow pipes causing a sharp compression in the fluid flow. This leads to disruption of the relatively large and polydisperse fat globules into a large number of lipid droplets that are much smaller and show a homogeneous size range. In total, these lipid droplets have a much larger surface area adsorb mainly caseins and to a minor extent whey proteins, resulting in fat globules loaded with protein. Conflicting data exist concerning the effect of homogenization on the allergenicity of milk proteins. Using a murine model, Poulsen and colleagues could show that increasing the fat contents in combination with homogenization resulted in an increase in the ability of the milk to induce anaphylactic reactions [71]. In line with this, a double-blind placebo-controlled study in milk allergic children revealed an increased ability of homogenized/pasteurized milk to evoke allergic reactions [72]. However, this could not be confirmed by other studies [73,74,75]. Further studies in humans did not show differences in the tolerance to homogenized and unhomogenized milk, respectively [76]. A review by Paschke et al. indicated that homogenization does not alleviate the potency of cow’s milk allergens [77].

#### 5.2.2. High Pressure Homogenization (HPH)

HPH (150 MPa) has been proposed as a substitute for the thermal processing of food. The binding capacity of casein to IgE before and after high pressure homogenization was reduced, as shown by ELISA [78]. However, more research is needed to determine the precise effect of homogenization on the allergenicity of milk allergens.

#### 5.2.3. Ultrasonic Treatment

Ultrasonic technology is used in food industry to improve food quality efficiently and also to develop new products with unique functions. Ultrasound improves the foaming and emulsifying properties and also casein stability [79] and can be done through a bath or an ultrasound probe. Two main differences exist when comparing these two possibilities: first, the ultrasonic probe is immersed directly into the solution, where the sonication takes place; and second, the ultrasonic power provided by the probe is much greater than the one supplied by the bath. The application of the high intensity focused ultrasound allows for the accelerated digestion of the proteins. This is of special interest when it comes to allergenicity. Depending on the intensity, different degrees of hydrolysis can be obtained. Decreased allergenicity has been reported for casein via colloid formation [80]. In line with this, a marked decrease in allergenicity was observed for β-lactoglobulin [81]. However, this technique has some disadvantages as it leads to the formation of free radicals [82].

#### 5.2.4. Enzymatic Processes

Enzymatic hydrolysis is a method used to break down proteins into smaller peptides or amino acids, resulting in a loss of structure and thus the removal of the conformational epitopes that are recognized by specific IgE. This method is applied to generate hypoallergenic milk formulas for milk allergic babies. These products contain only short peptides, which are unable to induce an allergic reaction. In a mouse model of sensitization, Duan and colleagues could show that mice sensitized by hydrolysates of β-lactoglobulin showed a significantly lower spleen lymphocyte proliferation level than those sensitized by intact β-lactoglobulin suggesting that enzymatic hydrolysis reduces its allergenicity [83]. Other studies showed retained or even enhanced allergenicity after enzymatic proteolysis. The digestion of milk proteins could also unmask epitopes leading to the increased binding of specific IgE. This disagreement is most likely due to the use of different enzymes [84,85]. Moreover, it was shown that peptides with a molecular mass smaller than 3 kDa remained allergenic [86]. It seems that choice of an appropriate enzyme to effectively decrease residual antigenicity is of great importance. Other studies showed that the combination of enzymatic digestion with heat-processing led to increased reduction of antigenicity [87,88]. The effect of enzymatic hydrolysis is reviewed in detail by [89].

The cross-linking of food proteins is often used to enhance food stability. Several highly specific enzymes (e.g., transglutaminase, horse radish peroxidase, laccase, tyrosinase) are used as additives in food industry to improve texture and functional properties. Currently, the only enzyme approved by the European Union for food marketing is transglutaminase. The treatment of whey proteins with transglutaminase seems to hide important epitopes, thus reducing their allergenic potential [90]. However, when using other enzymes, the treatment may also show unwanted effects such as increased allergenicity [91]. Another important factor is the pretreatment of proteins to make proteins accessible for enzymatic digestion. Chemical or thermal pretreatments are usually applied to improve the accessibility for enzymatic digestion. Therefore, all cross-linked proteins have to be tested for their capacity to bind specific IgE [92].

#### 5.2.5. Fermentation

During microbial fermentation, lactic acid bacteria (LAB) produce proteolytic enzymes causing the degradation of milk proteins to peptides and amino acids. This not only decreases the allergenicity of milk allergens due to the breakdown of IgE epitopes [93,94], but also leads to the production of bioactive peptides. The grade of the reduction in allergenicity is dependent on the proteases and peptidases of the LAB strains used [95]. Several studies exist showing increased tolerability of fermented milk products such as yogurt and cheese. Alessandri and colleagues showed that 58% of patients clinically reactive to cow’s milk tolerated fully maturated Parmigiano-Reggiano [96]. Yogurt is tolerated by the majority of children with cow’s milk allergy [97].

### 5.3. Novel Techniques

#### 5.3.1. Irradiation

Gamma irradiation has been proven to be an effective and safe method for the sterilization of certain products. However, this treatment does not only inhibit microorganisms but also alters the structure of the molecules targeted by this process. Thus, irradiation can improve the quality of milk, but can also introduce modifications. Beta-lactoglobulin (in solution) subjected to gamma radiation led to changes in the secondary and tertiary structure leading to protein aggregation [98]. Several studies have indicated that ionizing radiation could reduce allergenicity by the destruction of human IgE-binding epitopes in milk allergens depending on the dose of irradiation [99]. A study assessing the allergenicity of irradiated dairy products in BALB/c mice revealed that gamma irradiation influenced the epitopes of the major milk proteins and was associated with lower allergenicity of lyophilized irradiated milk [100]. In line with this, another study, investigating ultrasound-assisted irradiation for reducing the allergenicity of β-lactoglobulin, revealed that IgE binding capacity and release of inflammatory mediators from human basophil cells were reduced significantly [69].

#### 5.3.2. Microwave Treatment

Microwave treatment has been shown to be a good alternative to conventional heat treatments. Microwaves are electromagnetic waves and heat is generated following the absorption of microwave energy by water, organic molecules, or ions. However, phenomena that cannot be explained by the increase in temperature also occur and there is evidence that microwaves contribute to the existence of the nonthermal effects of microwave treatment [101]. Therefore, it is important to investigate changes in the structure and allergenicity of the proteins, especially if microwave treatment is performed in combination with other treatments. A study by Izquierdo and colleagues showed that microwave irradiation accelerates enzymatic treatments and also increases the degree of hydrolysis [87]. Another study, comparing the effects of microwave heating and conventional heating on the secondary and tertiary structures of β-lactoglobulin, revealed a substantial enhanced unfolding and exposure of buried amide groups. Thus, microwave processing could be a future alternative to produce hydrolysates with lower allergenicity [102,103].

#### 5.3.3. Cold Plasma Treatment

Atmospheric cold plasma appeared to be a promising novel technology to induce structural modifications of proteins [104]. It has been reported that treatment with cold plasma induces structural modifications and alters the antigenic response of the bovine milk allergens. In a recent study, casein, β-lactoglobulin and α-lactalbumin were analyzed before and after plasma treatment. The results revealed alterations in the secondary structure of the protein and decreased antigenicity of the casein and α-lactalbumin, whereas β-lactoglobulin showed increased antigenicity [105].

## 6. Concluding Remarks

For thousands of years, milk has been part of the human diet. In ancient times milk was consumed either raw or fermented, but since the late 19th century, milk has regularly been heat-treated to destroy pathogenic microorganisms to be safe for human consumption. In the recent past, milk has become more and more processed, not only for safety reasons but also to prolong shelf life and fulfil consumer expectations (convenient food products). However, intensive processing can also lead to the reduction of the bioactive compounds, which become degraded.

Cow’s milk also causes immune-mediated diseases such as non-IgE-mediated gastrointestinal food allergic disorders, which start early in life, and the total protein fraction seems to be a relevant trigger. Currently there is a lack of clear understanding of the different pathomechanisms. However, since most of these diseases resolve early in childhood, maturation and tolerance induction in the gastrointestinal tract may also be supportive of the resolution of the symptoms. IgE-mediated cow’s milk allergy also starts early in infancy and the majority of patients experience cure within 4–5 years, while a minority will also suffer from cow’s milk allergy in adulthood. Relevant IgE binding allergens have been identified and the diagnosis and treatment of cow’s milk allergy has improved due to our knowledge on the individual immunoactive proteins. However, there is only limited knowledge on how different processing methods affect the allergenicity of individual milk proteins. The thermal processing of proteins can affect their 3D structure and thus destroy IgE-binding epitopes or expose those that have been buried inside the structure. For whey proteins, different temperature ranges affect them, either up- or downregulating their allergenicity. This was also found for caseins, which showed different sensitivities for the individual caseins. The homogenization of milk results in a large number of lipid droplets adsorbing caseins and whey proteins. This can lead to an increase in allergic reactions in patients. Ultrasonic treatment facilitates colloid formation, which in turn can reduce allergenicity, as shown for β-lactoglobulin. It is generally accepted that enzymatic degradation breaks down proteins into peptides, resulting in hypoallergenic products. However, this mainly depends on the enzymes applied and the detailed analysis of the size and sequence of the obtained peptides. In the case of the cross-linking of food proteins via transglutaminase, epitopes may be hidden, as shown for whey proteins. Applying microbial fermentation for the production of yogurt and cheese can be beneficial for milk allergic patients since the allergens are degraded. For other processing techniques such as irradiation, microwave treatment, and cold plasma treatment, reports showed that these methods can affect the structure of the individual allergens and thus their allergenicity. However, at present only a low number of studies have been performed, with sometimes conflicting evidence. More evidence is needed to obtain a better understanding of which methods are relevant for reducing the allergenicity of the individual proteins present in milk, while preserving the bioactive ingredients of this precious food as part of the human diet.

## Figures and Tables

**Table 1:** Characteristics of main cow’s milk allergens [13,17] and IUIS Allergen Nomenclature.

Milk Proteins		Conc. (g/L)	Molecular Mass (kDa)	Biological Function	Amino Acid No.	Allergenic Activity *
**Whey proteins** (20% ≈ 7 g/L)	α-lactalbumin **Bos d 4**	1.2–1.5	14.2	Contributes to lactose synthesis	123	**Major**
	β-lactoglobulin **Bos d 5**	3–4	18.3	Binds to numerous hydrophobic and amphiphilic ligands (defined biological function still unclear)	162	**Major**
	BSA **Bos d 6**	0.4	67	Binds to fatty acids, flavors, metal ions	583	**Minor**
	Immunoglobulins **Bos d 7**	0.47	76.2	Antibacterial and antiviral activities		**Minor**
**Caseins** (80% ≈ 29 g/L) **Bos d 8**	αs1-casein **Bos d 9**	12–14	23.6	Calcium binding	199	**Major**
	αs2-casein **Bos d 10**	3.75–4	25.2	Calcium binding	207	**Minor**
	β-casein **Bos d 11**	10.5–12	24.0	Calcium binding	209	**Major**
	κ-casein **Bos d 12**	3.75–4	19.0	Stabilization and coagulation of milk	169	**Minor**

* Allergenic activity is presented as major versus minor allergen.

## Data Availability

Not applicable.
